# Stress-related expectations about smoking cessation and future quit attempts and abstinence - a prospective study in daily smokers who wish to quit^☆^

**DOI:** 10.1016/j.pmedr.2017.03.004

**Published:** 2017-03-18

**Authors:** Lise Skrubbeltrang Skov-Ettrup, Kia Kejlskov Egan, Peter Dalum, Janne Schurmann Tolstrup

**Affiliations:** a National Institute of Public Health, University of Southern Denmark, Oester Farimagsgade 5A, 1353 Copenhagen, Denmark; b Department of Cancer Prevention and Information, Danish Cancer Society, Strandboulevarden 49, 2100 Copenhagen, Denmark

**Keywords:** Stress, Smoking cessation, Quit attempts

## Abstract

Smokers who wish to quit may refrain from doing so if they expect to experience more stress after haven given up. We test if stress-related expectations about smoking cessation are associated with quit attempts and abstinence among smokers who are motivated to quit. The study included 1809 daily smokers in Denmark in 2011–2013. Stress-related expectations (do you think you will be more, less or equally stressed as a non-smoker?) were measured at baseline. Quit attempts, 30-day point prevalence abstinence and prolonged abstinence (defined as having been abstinent since baseline), were measured after 3, 8 and 14 months.

We found that the association between expecting to be more stressed if giving up smoking differed between participants who had previously attempted to quit and those who had not: In participants who previously attempted to quit (47%), expecting to be more stressed was associated with significantly lower odds of abstinence compared to smokers who expected the same or a lower level of stress (odds ratios were 0.49 (95% CI: 0.31–0.79) for 30-day abstinence and was 0.28 (95% CI: 0.08–0.99) for prolonged abstinence). In participants who had not previously attempted to quit, expectations about stress were not associated with abstinence.

Results indicate that expectations about stress in relation to smoking cessation are an important determinant of cessation in smokers who previously attempted to quit. Addressing stress and how to handle stressful situations may increase the likelihood of a successful quit attempt.

## 1 Introduction

Smoking cessation has been shown to be associated with stress reduction (Taylor et al., 2014). Nevertheless, stress relief is commonly perceived as an advantage of smoking and beliefs about smoking as a stress reducing agent are prevalent among smokers, even years after having quit (Herd and Borland, 2009). The sensation of nicotine withdrawal in many ways resemble the experience of stress, and relieving nicotine withdrawal by smoking may therefore be mistaken for general stress reduction (Carter et al., 2014).

Expectations about stress after smoking cessation are likely to affect the process of cessation. Social cognitive theory suggests outcome expectations to be important determinants of behaviour change (Bandura, 1998) and perceived pros and cons of quitting have been found to vary across stages in the smoking cessation process (Dijkstra et al., 1996). However, outcome expectations in relation to health and other gains have not been found to positively predict abstinence (Hyland et al., 2006, Li et al., 2011). Beliefs that smoking reduces negative affect have been found to predict smoking cessation (Wetter et al., 1994) and have also been found to mediate the association between perceived stress and perceived barriers to quitting (Robles et al., 2016).

In the present study, we test the hypothesis that expecting to be more stressed when quitting smoking is associated with fewer quit attempts and lower abstinence during a period of 14 months among men and women who are daily cigarette smokers and motivated to quit.

## 2 Material and methods

The study was based on data from a randomised controlled trial with the purpose to compare the effectiveness of proactive telephone counselling, reactive telephone counselling and an internet- and text-message-based intervention with a self-help booklet, on smoking cessation. Participants were assigned in equal ratio to all four groups. Participants were 1809 men and women who were daily smokers and motivated to quit (based on self-report of wanting to give up smoking at study inclusion). At study start (baseline), participants answered an online questionnaire concerning previous and current smoking habits, demographic variables and stress-related expectations. Follow-up by questionnaire was conducted 3, 8 and 14 months after baseline. The trial is described in detail elsewhere (Skov-Ettrup et al., 2016).

### 2.1 Stress-related expectations

Stress-related expectations were assessed using the following question: “As a non-smoker, do you think that you will be: *A lot less stressed*, *less stressed*, *no more or no less stressed*, *more stressed*, *a lot more stressed*”. We created a three categorical variable by combining the first two categories into “less level of stress”, and the two latter categories into “more stressed”.

### 2.2 Other variables

Participants gave information about number of previous quit attempts, usual number of cigarettes per day, self-efficacy and Fagerström Test for Nicotine Dependence (FTND). Additionally, information about age and educational status (ISCED; International Standard Classification of Education) was collected at baseline.

### 2.3 Outcomes

Outcomes included quit attempts, 30-day point prevalence abstinence and 12 months prolonged abstinence. At the three follow-ups, participants were asked to indicate the number of times they attempted to quit since their inclusion in the study. 30-day point prevalence abstinence was defined as having been abstinent for at least 30 days and was measured at 3, 8 and 14 months. 12 months prolonged abstinence was measured at the 14 months follow-up.

### 2.4 Statistical analysis

The association between stress-related expectations and smoking cessation outcomes (quit attempts, 30-day abstinence and prolonged abstinence) was analysed by logistic regression, separately at each follow-up (3, 8 and 14 months), and combined in a repeated measurements model. In the repeated model, it was initially tested if associations between stress-related expectations about smoking cessation and smoking cessation outcomes varied at the three time points. This was done by a nested log-likelihood test, comparing a model containing main effects of stress-related expectations and of time, with a model also including interaction terms. In all tests, *p*-values were > 0.05 and interaction terms were excluded from the final model, allowing for the estimation of the association between stress-related expectations and quitting outcome at all three follow-ups by a single parameter (odds ratio). By similar principle, it was tested if there was interaction between stress-related expectations and type of intervention, to clarify if any of the interventions reduced or eliminated the effects of expected stress on cessation outcomes. This was, however, not the case (*p*-value for interaction > 0.05).

Odds-ratios were adjusted for sex, age, education, nicotine dependence (FTND) and intervention group. We also performed analyses counting non-responders as smokers (intention to treat analysis). All analyses were performed using Stata 13.1.

## 3 Results

In total, 1809 daily smokers participated. Response rates at 3, 8 and 14 months follow-ups were 78%, 82% and 80%, respectively. The majority of smokers believed that they would experience the same level of stress as ex-smokers (64%) whereas 23% expected to be more and 13% expected to be less stressed.

Higher age and lower self-efficacy was associated with the expectation to experience more stress if quitting, whereas previously having attempted to quit was more prevalent in participants who expected to experience less stress if quitting (Table 1). All over, 846 (47%) participants had previously attempted to quit one or more times.

Stress-related expectations about smoking cessation were not associated with future attempts to quit smoking, at all time points or in repeated measurements model (Table 2).

For 30-day point prevalence abstinence and prolonged abstinence, we observed statistically significant interactions between previously having attempted to quit and stress-related expectations about smoking cessation (*p*-values for interaction < 0.05): In participants who had not attempted to quit previously, stress-related expectations were not associated with 30-day point prevalence abstinence or with prolonged abstinence. In participants who previously attempted to quit, expecting to be more stressed as a future non-smoker was consistently associated with lower odds of 30-day point prevalence abstinence and prolonged abstinence at all three time points, and in repeated measurements models. For instance, odds ratios of 30-day point prevalence abstinence was 0.49 (0.31–0.79) for 30-day abstinence and 0.28 (0.08–0.99) for prolonged abstinence in those who expected that they would be more stressed as non-smokers compared to those expecting the same or lower level of stress (Table 3, repeated measurements models, intention to treat analysis).

Of the potential confounders included in the model, long education (15 + years), lower score on FTND and the proactive telephone counselling intervention were statistically significantly associated with smoking cessation.

Lastly, sensitivity analyses were performed, including self-efficacy in adjusted models. As expected, higher self-efficacy was independently associated with odds ratio of quitting smoking. For each higher score of self-efficacy, the odds ratios of 30-day abstinence and prolonged abstinence were 1.02 (95% CI: 1.01–1.03) and 1.03 (95% CI: 1.01–1.05). However, adjusting stress-related expectations about smoking cessation for self-efficacy had little effect. For instance, the adjusted odds ratio or 30-day point prevalence abstinence was 0.49 (0.31–0.79) for 30-day abstinence and was 0.52 (0.32–0.85) after including self-efficacy, in those who expected that they would be more stressed as non-smokers compared to those expecting the same or lower level of stress.

## 4 Discussion

In this study, we found that 23% of the participants who were daily smokers expected to become more stressed as non-smokers. This belief was associated with lower chance of smoking cessation during a period of 14 months in participants who had previously attempted to quit. In participants who attempted to quit for the first time, expecting to be more stressed as a non-smoker was not associated with smoking cessation.

Interestingly, expecting to be more stressed as a non-smoker associated differently with smoking cessation in smokers with and without previous quit attempts. In contrast to individuals who have not previously attempted to quit, individuals who already have tried to quit, have experience with quitting and thus with stress related to this process. It has been shown that the ability to calm down when feeling stressed or upset is perceived to be worse during the early stages of a quit attempt, but improves as time since quitting increases (Yong et al., 2010). Furthermore, unsuccessful quit attempts may induce stress (Manning et al., 2005). Consequently, having experienced failed quit attempts as stressful could result in negative expectancies about future quit attempts, especially if the previous attempts of cessation were short-term.

Negative reinforcement expectancies i.e. beliefs that smoking reduces negative affect are inversely associated with abstinence from smoking (Wetter et al., 1994). This concept has also been found to mediate the association between perceived stress and barriers to quitting (Robles et al., 2016). Our results indicate that stress related expectations about smoking cessation play a similar role.

Some limitations are important to consider in the present study. The measure of stress-related expectancies about smoking cessation was not validated. The results could, furthermore, have been confounded by participants' actual level of stress which was not measured. Also, smoking status was not biochemically validated which implied a risk of smoking cessation being overestimated. Since the interventions and data collection did not involve any face to face contact biochemical validation was not feasible. Around 20% of participants were lost to follow-up. This could have introduced bias if non-responders differed from responders and the association between smoking related expectations and smoking status differed for responders and non-responders. Missing information on quit attempts and 30-day point prevalence abstinence was imputed using simple imputation, by counting non-responders as smokers as suggested for outcome data in randomised controlled trials for smoking cessation. Results were similar, thus, indicating that the results were relatively robust with regards to selection bias caused by loss to follow-up.

In conclusion, expectations about stress in individuals who previously attempted to quit smoking unsuccessfully may be an important determinant for smoking cessation later on. Addressing stress and how to handle stressful situations may increase the likelihood of a successful quit attempt among smokers who are motivated to quit.

## Figures and Tables

**Table 1:** Baseline characteristics by expectations about stress associated with quitting smoking. Denmark 2011–2013 (n = 1809).

	Expectations about stress if quitting			
	Less stressed (n = 241, 13%)	Same level of stress (n = 1152, 64%)	More stressed (n = 416, 23%)	P for trend
Men, n%	76 (32)	496 (43)	180 (43)	0.01
Age, mean (SD)	46.1 (12.4)	50.6 (12.5)	52.2 (12.8)	< 0.0001
Education, short, n%	33 (14)	153 (13)	66 (16)	0.33
Previous quit attempts (%≥1)	154 (64)	503 (44)	189 (45)	< 0.0001
Cigarettes/day, mean (SD)	16.6 (7.1)	16.5 (8.2)	17.1 (8.4)	0.56
Nicotine dependence, FTND^a^ score, mean (SD)	4.7 (2.3)	4.2 (2.3)	4.6 (2.2)	0.78
Self-efficacy, mean (SD)	24.3 (12)	24.2 (11)	22.2 (10)	0.009

a Fagerström Test for Nicotine Dependence.

**Table 2:** Odds-ratios^a^ (95% confidence intervals) for attempting to quit by baseline expectations about stress if quitting. Denmark 2011–2013 (n = 1809).

	N outcomes/N for analysis	Less stressed (n = 241)	Same level of stress (n = 1152)	More stressed (n = 416)
Quit attempts				
3 month	770/1416	0.80 (0.57–1.12)	1.00 (ref)	0.80 (0.61–1.05)
8 month	1128/1463	0.85 (0.58–1.27)	1.00 (ref)	0.83 (0.61–1.12)
14 month	971/1351	0.99 (0.67–1.48)	1.00 (ref)	1.91 (0.68–1.23)
Repeated measurements model^b^	2869/4230	0.87 (0.67–1.13)	1.00 (ref)	0.85 (0.69–1.05)
Repeated measurements model, ITT^b^, ^c^	2869/5427	1.01 (0.82–1.25)	1.00 (ref)	0.88 (0.74–1.05)

a Adjusted for age, sex, education, nicotine dependence (Fagerström Test for Nicotine Dependence), previous quit attempts and intervention group.

b Repeated measurements model with outcomes measured 3, 8 and 14 months after baseline, adjusted for a and time (3, 8, 14 months).

c ITT – intention to treat analysis, treating non-respondents as smokers.

**Table 3:** Odds-ratios^a^ (95% confidence intervals) for 30-day point prevalence abstinence and 12 months prolonged abstinence according to baseline expectations about stress if quitting. Denmark 2011–2013, quit attempters only (n = 1398).

		Expectations about stress if quitting					
		No previous quit attempts, (n = 673, 48%)			Previous quit attempts, (n = 725, 52%)		
	N outcomes/N for analysis	Less stressed (n = 87)	Same level of stress (n = 649)	More stressed (n = 227)	Less stressed (n = 154)	Same level of stress (n = 503)	More stressed (n = 189)
30-day abstinence^d^							
3 month	185/770	1.07 (0.46–2.47)	1.00 (ref)	1.15 (0.64–2.08)	1.23 (0.65–2.33)	1.00 (ref)	0.53 (0.25–1.11)
8 month	193/1099	0.71 (0.31–1.61)	1.00 (ref)	1.05 (0.62–1.80)	0.70 (0.37–1.33)	1.00 (ref)	0.36 (0.17–0.76)
14 month	300/971	0.47 (0.22–0.98)	1.00 (ref)	0.82 (0.51–1.33)	0.84 (0.49–1.44)	1.00 (ref)	0.59 (0.34–1.03)
Repeated measurements model^b^	678/2840	0.72 (0.41–1.26)	1.00 (ref)	0.90 (0.61–1.35)	0.83 (0.55–1.25)	1.00 (ref)	0.50 (0.31–0.81)
Repeated measurements model, ITT^b^, ^c^	678/2869	0.82 (0.48–1.42)	1.00 (ref)	0.91 (0.61–1.34)	0.87 (0.58–1.30)	1.00 (ref)	0.49 (0.31–0.79)

Prolonged abstinence^d^							
8 month	99/610	0.68 (0.21–2.18)	1.00 (ref)	0.99 (0.47–2.10)	0.56 (0.20–1-57)	1.00 (ref)	0.21 (0.05–0.90)
14 month	81/494	0.58 (0.15–2.27)	1.00 (ref)	0.99 (0.45–2.20)	0.62 (0.20–1.94)	1.00 (ref)	0.27 (0.06–1.20)
Repeated measurements model^b^	180/2070	0.63 (0.21–1.89)	1.00 (ref)	1.15 (0.61–2.21)	0.50 (0.19–1.39)	1.00 (ref)	0.27 (0.08–0.98)
Repeated measurements model, ITT^b^, ^c^	180/2099	0.63 (0.21–1.89)	1.00 (ref)	1.15 (0.60–2.19)	0.51 (0.18–1.41)	1.00 (ref)	0.28 (0.08–0.99)

a Adjusted for age, sex, education, nicotine dependence (Fagerstöm Test for Nicotine Dependence) and intervention group.

b Repeated measurements model with outcomes measured 3, 8 and 14 months after baseline, adjusted for a and time (3, 8, 14 months).

c ITT – intention to treat analysis, treating non-respondents as smokers.

d In participants who reported to having attempted to quit.
